# A novel NAA10 p.(R83H) variant with impaired acetyltransferase activity identified in two boys with ID and microcephaly

**DOI:** 10.1186/s12881-019-0803-1

**Published:** 2019-06-07

**Authors:** Rasmus Ree, Anni Sofie Geithus, Pernille Mathiesen Tørring, Kristina Pilekær Sørensen, Mads Damkjær, DDD study, Sally Ann Lynch, Thomas Arnesen

**Affiliations:** 1 Department of Biomedicine, University of Bergen, Jonas Lies vei 91, NO-5020 Bergen, Norway; 2 Department of Clinical Genetics, Odense University Hospital, DK-5000 Odense C, Denmark; 3 Hans Christian Andersen Children’s Hospital, Odense University Hospital, DK-5000 Odense C, Denmark; 4 DDD Study, Wellcome Trust Sanger Institute, Hinxton, Cambridge, UK; 5 Temple Street Children’s Hospital, Temple Street, Dublin, D01 X584 Ireland; 6 Department of Biological Sciences, University of Bergen, NO-5020 Bergen, Norway; 7 Department of Surgery, Haukeland University Hospital, NO-5021 Bergen, Norway

**Keywords:** NAA10, X-linked, XLID, Microcephaly, Intellectual disability, N-alpha-acetyltransferase, Acetylation, NatA

## Abstract

**Background:**

N-terminal acetylation is a common protein modification in human cells and is catalysed by N-terminal acetyltransferases (NATs), mostly cotranslationally. The NAA10-NAA15 (NatA) protein complex is the major NAT, responsible for acetylating ~ 40% of human proteins. Recently, *NAA10* germline variants were found in patients with the X-linked lethal Ogden syndrome, and in other familial or de novo cases with variable degrees of developmental delay, intellectual disability (ID) and cardiac anomalies.

**Methods:**

Here we report a novel *NAA10* (NM_003491.3) c.248G > A, p.(R83H) missense variant in NAA10 which was detected by whole exome sequencing in two unrelated boys with intellectual disability, developmental delay, ADHD like behaviour, very limited speech and cardiac abnormalities. We employ in vitro acetylation assays to functionally test the impact of this variant on NAA10 enzyme activity.

**Results:**

Functional characterization of NAA10-R83H by in vitro acetylation assays revealed a reduced enzymatic activity of monomeric NAA10-R83H. This variant is modelled to have an altered charge density in the acetyl-coenzyme A (Ac-CoA) binding region of NAA10.

**Conclusions:**

We show that NAA10-R83H has a reduced monomeric catalytic activity, likely due to impaired enzyme-Ac-CoA binding. Our data support a model where reduced NAA10 and/or NatA activity cause the phenotypes observed in the two patients.

**Electronic supplementary material:**

The online version of this article (10.1186/s12881-019-0803-1) contains supplementary material, which is available to authorized users.

## Background

N-terminal acetylation (Nt-acetylation) is an exceedingly prevalent protein modification, affecting an estimated 80% of human proteins [1]. An acetyl group donated by acetyl coenzyme A (Ac-CoA) is attached to the N-terminal amine group of a substrate protein through the catalytic action of N-terminal acetyltransferases (NATs), which are members of the GCN5-related N-acetyltransfererase (GNAT) superfamily [2]. Nt-acetylation may affect substrates in several ways, although for most substrates the importance of the Nt-acetyl group remains enigmatic. Nt-acetylation is reported to affect the half-life of substrates, destabilizing some substrates [3–5] and stabilizing others [6, 7]. Nt-acetylation also regulates folding [8–11], protein complex formation [12–17], subcellular localization and membrane interaction [16–19]. While several post-translational NATs have recently been described [20–25], most substrates appear to be Nt-acetylated at the ribosome by one of at least five ribosome-associated NATs – NatA, NatB, NatC, NatD, or NatE. Chief among these in terms of substrate diversity is NatA [1]. The human NatA complex is composed of the catalytic subunit NAA10 and the auxiliary subunits NAA15, NAA50 and HYPK [11, 26–29]. The ribosome-associated NatA complex Nt-acetylates an estimated 40% of the human proteome [30], preferring small, polar amino acids which are exposed after initiator methionine excision [26]. NAA10 is mainly found in complex with NAA15, both ribosome-and non-ribosome associated [31, 32]; however, there also exists a pool of non-NAA15 bound NAA10 [31]. Due to a conformational change in NAA10 upon NAA15 association, NAA10 alone has an altered substrate specificity, preferring acidic N-termini [31, 33]. Further, non-NAT functions of NAA10 as a lysine acetyltransferase (KAT) or as a non-catalytic regulator have been proposed [34–38]. Loss of NAA10 leads to developmental defects or embryonic death in several model organisms [34, 39–41]. Hereditary or de novo germline variants in the X-chromosomal *NAA10* gene is associated with developmental syndromes and non-syndromic developmental delay in humans. A NAA10 S37P missense mutation is the cause of the Ogden syndrome, an extremely rare disease in which affected boys have an aged appearance, craniofacial anomalies, cardiac problems including arrhythmia, and where all affected boys have died by age 16 months [42]. This mutation affects NatA complex formation and leads to lowered cell proliferation, larger cell size and reduced Nt-acetylation of some NatA substrates [6, 42]. One splice-donor mutation was found to lead to Lenz microphthalmia syndrome, causing small or missing eyes, intellectual disability and skeletal, cardiac, and renal problems [43]. Various other mutations lead to non-syndromic developmental delay and seizures in males and females [44, 45], a novel intellectual disability syndrome in two brothers carrying the same mutation [46], intellectual disability, developmental delay and cardiac abnormalities in three brothers from two families [47], and non-syndromic intellectual disability with delayed language and motor development in a female proband [48]. The NAA10 c.247C > T p.R83C missense mutation is recurrent, previously having appeared de novo in one male and seven female patients, generally manifesting with moderate to severe intellectual disability and developmental delay, though only the boy had EEG anomalies [49]. While NAA10 mutations have a heterogenous clinical picture, with no clear genotype-catalytic activity-phenotype correlation [47], some features are seen in many or most patients; intellectual disability, developmental delay, growth failure, and cardiac anomalies. Here, we present the c.248G > A p.R83H variant, found in two boys, aged 15 and 12 with hyperactivity, limited language development, developmental delay, intellectual disability and hypertrophic cardiomyopathy. The NAA10 R83H mutation leads to a substantial decrease in NAA10 catalytic activity, supporting the hypothesis that this variant causes a loss of NAA10-mediated acetylation and is the cause of the observed phenotypes. Based on structural models of the variant, we predict that this reduced catalytic activity is due to impaired Ac-CoA binding.

## Methods

### Trio exome sequencing

A trio-based whole-exome sequencing approach was undertaken. For patient 1, whole exome sequence was performed as described [50]. The *NAA10* variant was verified by targeted Sanger sequencing. DNA from patient 2 and parents were subjected to exome capture using NimbleGen SeqCap EZ MedExome (Roche), followed by sequencing on an Illumina NextSeq550 to a mean coverage of 91x, with 94% of targeted bases covered with minimum 20x coverage. Raw reads were aligned using the Burrows-Wheeler Alignment tool (BWA-MEM) v. 0.7.15 [51] and the GATK Best Practice pipeline v. 3.8–0 was used for variant calling [52]. Annotation and filtering of variants was performed using VarSeq 1.5.0 (Golden Helix). The *NAA10* variant was verified by targeted Sanger sequencing. Informed consents were obtained from patient indexes and family members.

### Multiple sequence alignment, conservation scores and structural model

Multiple sequence alignments were created using ClustalX [53] and the illustration and conservation scores using Jalview [54]. The input sequences were: human NAA10 (Uniprot ID: P41227), *Mus musculus* Naa10 (Uniprot ID: Q9QY36), *Rattus norvegicus* Naa10 (Uniprot ID: D3ZUQ2), *Xenopus laevis* naa10 (Uniprot ID: Q7ZXW3), *Danio rerio* naa10 (Uniprot ID: Q7T3B8) and *Saccharomyces cerevisia* ARD1 (Uniprot ID: P07347). The NAA10 structural model is from the human NatA complex (PDB ID: 6C9M) [55]. The image was obtained by aligning this structure to the structure of *Schizosaccharomyces pombe* (4KVM) [33], which was solved with CoA and the substrate peptide SASE. The structure alignment and figure generation was performed using PyMol [56].

### In silico mutagenesis

NAA10-WT and NAA10-R83H from the human NatA complex (PDB ID: 6C9M [55]) were aligned in PyMOL to NAA10 from the *S*. *pombe* NatA complex (PDB ID: 4KVM [33]). In order to calculate the electrostatic potential of the two variants, these chains were uploaded to the Adaptive Poisson-Boltzmann Solver (APBS) PDB2PQR webserver [57–59] PDB2PQR makes preparatory changes to the PDB file by optimizing hydrogen bond, repairing heavy atoms, assessing pKa values and assigning charge and radius parameters [59]. The resulting PQR file was then solved for continuum electrostatic potential by the APBS software. Output files from the APBS were visualized in PyMOL and the chains were aligned. Both PDB files were prepared and calculated with the same parameters, optimized for cellular conditions. The molecular mechanical forcefield, Amber [60] and the heuristic pKa calculation software PROPKA [61] were used to generate the PQR at pH 7.4. Following the PQR preparations, APBS was set to utilize a manually-configured multigrid calculation, where the automatically suggested dimensions were applied. “Ionic strength of the solvent” was set to that of 0.15 M NaCl with ion radius of 1.5 Å. No electrostatic energies were calculated, and the output was set to “write out the electrostatic potential in units of k_b_T/e_c_ (multigrid and finite element)”. The APBS was then launched with the remaining parameters unchanged. The output PQR file from the APBS was then opened in PyMOL and visualized in surface view.

### Preparation of plasmids

In order to study the NM_003491.3 c.248G > A, p.(R83H) missense variant, a bacterial expression vector pETM-41/His-MBP-*NAA10* was modified by site-directed mutagenesis (Q5**®** Site Directed Mutagenesis Kit, New England Biolabs) according to the manufacturer’s protocol. The primers used for mutagenesis were NAA10 G248A p.R83H fwd (5′-CAACCTCCTCGGCCTGCCCGA) and NAA10 G248A p.R83H rev (CAGTGCTGCATGTTCATTAGGTC), with an annealing temperature of 69 °C. The plasmid was verified by sequencing.

### Protein expression in *E.coli* BL21 cells

To study the impact of the novel variant on in vitro catalytic activity, a two-step purification of His/MBP-NAA10-WT and His/MBP-NAA10-R83H expressed in BL21 Star DE3 *E. coli* was carried out essentially as described [44].

### In vitro colorimetric acetylation assays

The catalytic activity of the novel NAA10-R83H variant was compared with NAA10-WT by performing both time dependent and substrate dependent DTNB-based acetylation assays. In the substrate dependent assay, 50 μL reactions containing 100 nM His-MBP-NAA10-WT or NAA10-R83H, 300 μM peptide substrate (either EEEIA, DDDIA, MLGPE or SESSS, short for **EEEIAAL**RWGRPVGRRRRPVRVYP,**DDDIAAL**RWGRPVGRRRRPVRVYP, **MLGPEGG**RWGRPVGRRRRPVRVYP, and **SESSSKS**RWGRPVGRRRRPVRVYP, where the part in bold is the variable N-terminus and the rest of the amino acid sequence is identical between the peptides), 300 μM Ac-CoA, 1 x acetylation buffer (50 mM Tris-HCl, 1 mM EDTA and 10% glycerol, pH 8.5), incubated for 20 min at 37 °C. Blank reactions incubated in the absence of enzyme. The reactions were stopped using 100 μL quenching buffer (3.2 M guanidine-HCl, 100 mM Na_2_HPO_4_, pH 6.8), at which time the blanks were added enzyme in equal proportion to the reactions. To indicate the degree of acetylation for the individual reactions, 25 μL saturated solution of DTNB in DTNB buffer (100 mM Na_2_HPO_4_, 10 mM EDTA, pH 6.8) was added to each reaction. The time dependent assay was carried out using only EEEIA as a substrate, and reactions were quenched at different times (after 10, 20 and 30 min); the procedures for the assays were otherwise the same. Absorbance was measured at 412 nm using TECAN Infinite® 200 PRO plate reader.

## Results

### Description of patients

Patient 1 is the only child born to non-consanguineous parents. His birth weight was 3.25 kg (25th centile). There were no antenatal or neonatal complications. His father has microcephaly with mild learning difficulties; his mother attended mainstream school but left school early. Patient 1 presented with developmental delay at age 2. Early milestones: he sat at 9 month, walked age 2 years 2 months, he had very poor speech development, only has ~ 300 words with 2–3 worded sentences aged 15 years. He was diagnosed with coeliac disease aged 5 years 5 months (his father also has coeliac disease). He had short stature but this resolved once treated for coeliac disease. He has transient neutropenia of infancy which resolved. He developed epilepsy aged 13 years 10 months. He wears glasses for astigmatism. He has a history of chronic constipation. He has behavioural issues including poor concentration, and can be quite volatile and aggressive. He has been assessed as having a moderate learning disability & attends a special school. His cardiac assessment revealed a normal cardiac examination aged 2 years 9 months and again 15 years with a normal long QT interval. However, he had been noted to have mild concentric left ventricular hypertrophy on an assessment in between. He is hypermobile. He takes sodium valproate for epilepsy and movicol for his constipation. At 2 years 1 month of age his weight was 11.4 kg (9th centile)/height 80.5 cm (> 0.4th centile), and 24.6 kg (>3rd centile)/height 124.4 (> 0.4th centile) cm at 10 years 7 months. Occipitofrontal circumference (OFC) aged 14 years 7 months 52.5 cm (<10th centile) (His father’s OFC is 53.5 cm (< 0.4th centile). He has a tented upper lip but no other dysmorphism. Trio exome revealed a maternally inherited *NAA10* (NM_003491.3) c.248G > A, p.R83H variant which was further confirmed by Sanger sequencing. The mother’s parents are both dead precluding further segregation studies; however her two healthy brothers tested negative for this variant.

**Fig. 1:**
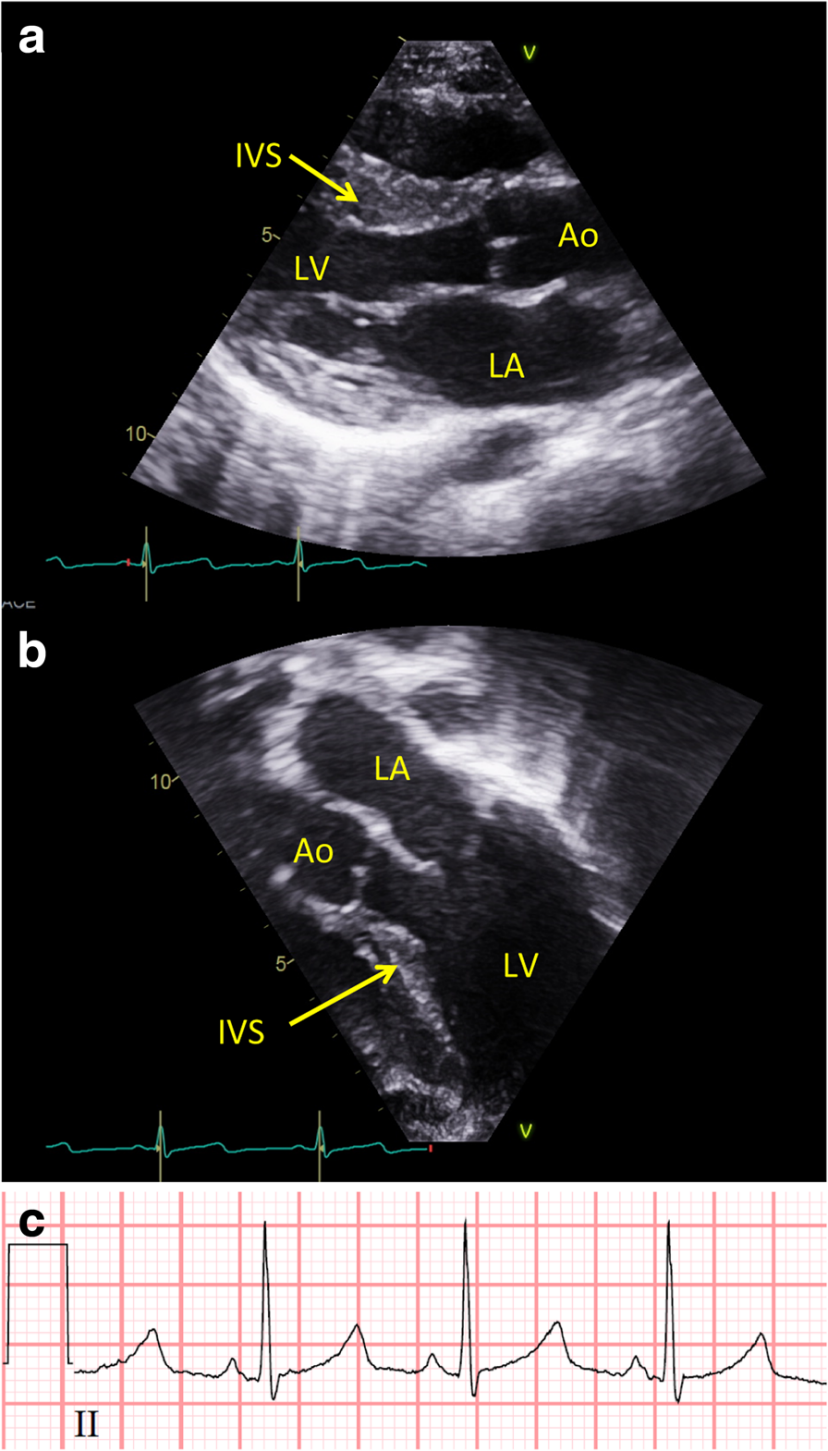
Cardiac hypertrophy in patient 2. Transthoracic echocardiography showing the left ventricle in **a** parasternal long axi and **b** apical 3-chamber view. Please note how the hypertrophy predominantly affects the interventricular septum with relative sparring of the apical and posterior regions. Red marker on EKG trace shows temporal relation to heart cycle. LV, left ventricle; Ao, aorta; LA, left atrium; IVS, interventricular septum. **c** Standard electrocardiogram (EKG) in patient 2. Note the borderline prolonged QTc

Patient 2 is the second child to non-consanguineous parents. He was born after 39 weeks of gestation. Birth weight 3344 g (median), length 51 cm (median), and head circumference 36 cm (+1SD). He had neonatal-onset hypotonia and poor feeding. He was breastfed to some extent until he was five years old. He presented with developmental delay at 12 months. Early milestones: he sat at 6 month, walked at age 2 years 3 months, from 18 months he rolled around indoors. He had very poor speech development and only uses a limited number of words with 2–3 worded sentences aged 12 years. He has behavioural issues including poor concentration, but is a quite happy and very active boy. He had eczema and very fine and sparse scalp hair until 3 years of age. He has a tendency to develop mild fevers of unknown origin. He does not have epilepsy. His height has been constantly at -2SD since 2 years of age; at 12 years his height was 142 cm. Dysmorphic features at 12 years of age was rather mild in the form of large ears, and rather closely spaced eyes. Cardiac examination revealed a structurally normal heart, i.e. no congenital malformations. Measurements of ventricular wall thickness were taken at end-diastole, indexed to body surface area and z-score measurements calculated as described by the Pediatric Heart Network [62]. This revealed a predominantly septal hypertrophy (z-score + 8.3) and reduced end-diastolic diameter of the left ventricle (Fig. 1). Although the septal hypertrophy caused narrowing of the left ventricular outflow tract, it did not cause obstruction to flow (Fig. 1 A and B, Additional file 1: Video S1). The ECG was normal except for borderline prolongation of the corrected QT-interval (420 milliseconds, Fig. 1 C). To assess for potential arrhythmias a 48-h Holter monitoring was performed. The heart rate varied between 66 to 165 bpm, and on average was 106 bpm. No arrhythmias were detected. Trio exome sequencing revealed a mosaic de novo NAA10 ((NM_003491.3) c.248G > A, p.(Arg83His)) hemizygote missense variant, with a mosaic degree of 75%. The variant was confirmed by Sanger sequencing.

### Functional testing

**Fig. 2:**
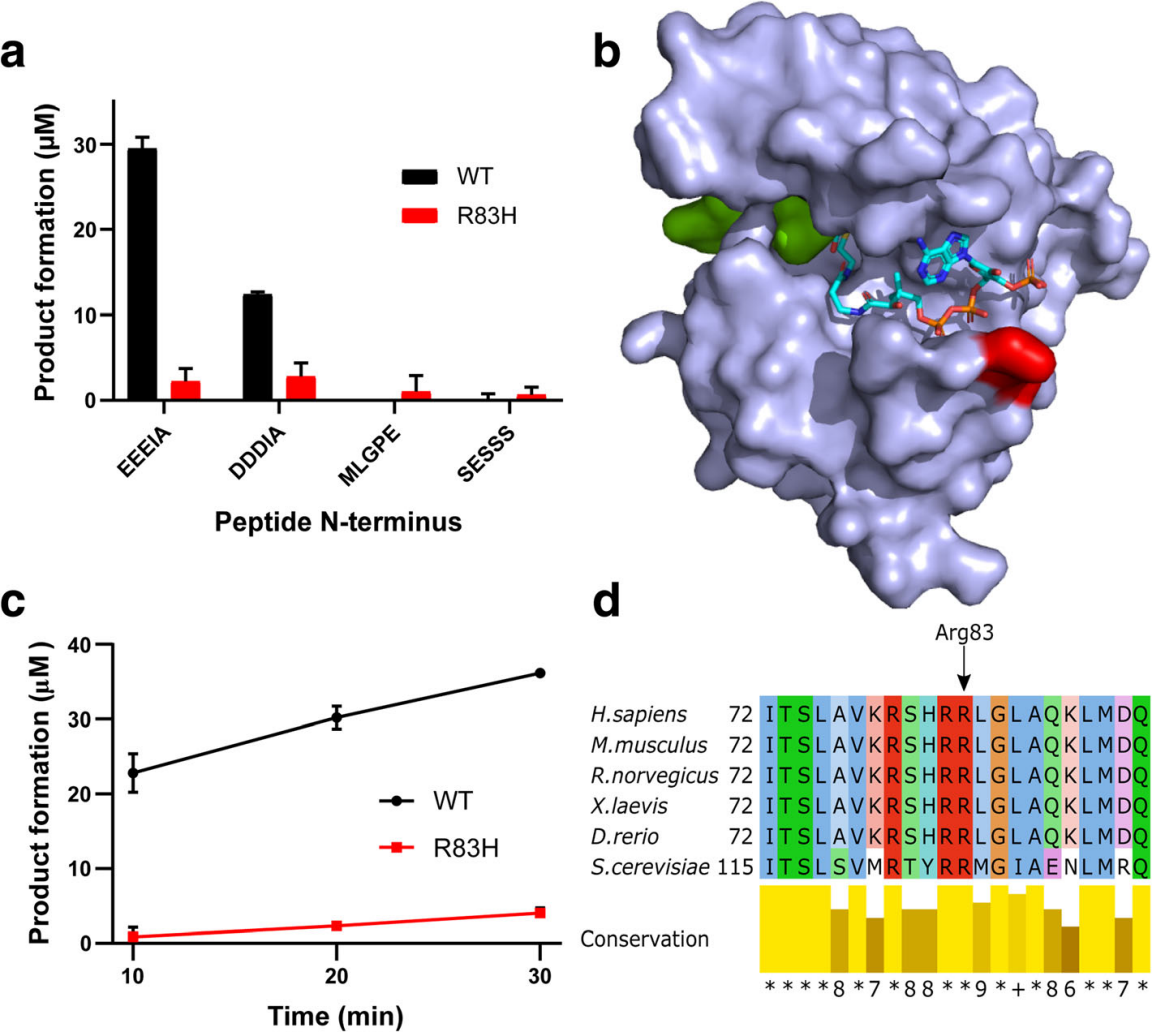
Functional testing of NAA10-R83H. MBP-NAA10 fusion proteins (wild-type (WT) or R83H) were tested for in vitro NAT activity against substrate peptides with indicated N-termini **a** or against EEEIA-peptide for the indicated reaction times **c**. Sequences of the peptide substrates are **EEEIAAL**RWGRPVGRRRRPVRVYP, **DDDIAAL**RWGRPVGRRRRPVRVYP, **MLGPEGG**RWGRPVGRRRRPVRVYP, and **SESSSKS**RWGRPVGRRRRPVRVYP (bold indicating the variable N-termini of the peptides, while the rest of the peptide sequence is identical between them). **b** Structure of the human NAA10 protein (PDB ID: 6C9M) superimposed on the substrate from the *Schizosaccharomyces pombe* NAA10 structure (4KVM) Substrate peptide (SASE) is shown in green, while the mutation site Arg83 is shown in red. CoA is shown as a licorice model colored according to element. **d** Multiple sequence alignment of NAA10 from *Homo sapiens*, *Mus musculus, Rattus norvegicus, Xenopus laevis, Danio rerio*, and *Saccharomyces cerevisiae*. Conservation score is calculated by Jalview (http://www.jalview.org/), with * signifying perfect conservation

To assess the effect of the R83H mutation on NAA10 catalytic activity, we performed site-directed mutagenesis to obtain the R83H mutant NAA10 protein. We expressed wild-type NAA10 (NAA10-WT) and NAA10-R83H as 6xHis-tagged maltose-binding protein (MBP)-fusion proteins in *E. coli*, purified them, and performed in vitro Nt-acetylation assays against model substrate peptides. These peptides have identical C-termini but differ in the 7 N-terminal amino acids. Such setups are commonly used to determine the substrate preferences of NATs, and to measure catalytic activities of NAT mutant enzymes [35, 48, 49]. We were able to purify NAA10-R83H in monomeric form under the same conditions as NAA10-WT. Both WT and R83H eluted at around 80 mL, and the R83H mutant was at the expected size*.* In vitro, monomeric NAA10, not bound to the NatA complex, prefers substrates with acidic N-termini [31], represented in our panel by EEEIA and DDDIA. The NatA canonical substrate SESSS and the NatE substrate MLGPE were also included. We found no difference in the preference for different peptides between NAA10-WT and NAA10-R83H, but the catalytic efficiency was severely diminished in the R83H mutant (Fig. 2a). Timecourse experiments with the EEEIA peptide confirmed that the catalytic activity is significantly reduced (Fig. 2c).

### Structural conservation and surface charge of NAA10-R83H

**Fig. 3:**
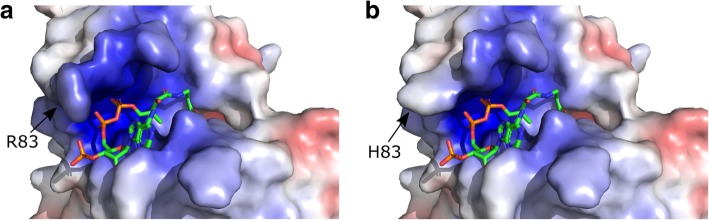
NAA10-R83H has altered charge distribution at the surface facing Ac-CoA WT R83 **a** or H83 mutant NAA10 **b** is shown in a surface model, with residue 83 marked with an arrow. Charge was visualized in PyMOL. Blue denotes positive charge and red denotes negative charge. Ac-CoA is shown as a licorice model

A structural model was made by aligning the human NAA10 chain from the structure of the human NatA complex (PDB ID: 6C9M, [55]) to the corresponding chain of the *S. pombe* (PDB ID: 4KVM, [33]), the latter of which was solved with CoA and substrate peptide. This showed that the mutation site R83 is in proximity to the negatively charged phosphate group on the ribose ring of CoA (Fig. 2b). A defining feature of GNAT acetyltransferases is a core fold which includes an Ac-CoA binding region, four alpha-helices and six or seven beta-sheets [1]. R83 is part of the Ac-CoA binding region of NAA10, though R82 is even more conserved (Fig. 2d). We find an arginine in the same position in crystal structures of NAA20 [63], NAA40 [64] NAA50 [65], and NAA60 [66]. At physiological pH, arginine is expected to be protonated to a greater degree than histidine. We performed in silico mutagenesis in NAA10 from the hNatA structure [55] to determine whether the Ac-CoA binding region had altered electrostatic properties (Fig. 3), and found that the surface near Ac-CoA appears less basic when R83 is mutated into a histidine.

## Discussion

NAA10 is predicted to Nt-acetylate around 40% of the human proteome [1] as part of the NatA complex on the ribosome [29], but it may also be a monomeric NAT enzyme not associated with the ribosome [31]. In addition, both lysine acetyltransferase activities and acetyltransferase-independent functions have been proposed for NAA10 [34–38]. NAA10 is essential for normal development in all surveyed model organisms [34, 39–41]. Several mutations in human NAA10 are known, with substantial heterogeneity in presentation and severity of symptoms, yet with the common features of intellectual disability, delayed developmental and growth failure, and occasionally cardiac anomalies [42–44, 46–49, 67, 68]. In many cases, NAA10 mutations result in a decrease in catalytic activity (S37P, Y43S, I72T, R83C, V107F, V111G, R116W, and F128 L), while others destabilize NAA10 (Y43S, I72T, V111G, F128I, F128 L). The mutation causative of Ogden syndrome, S37P, leads to impaired NAA10 interaction with other NatA complex components [6], in addition to having decreased catalytic activity [42]. A new potential mechanism for how NAA10 mutation may cause developmental phenotypes is acetyltransferase-independent, however. Three previously described variants (S37P, V107F and R116W) were found to have decreased binding to imprinting control regions, potentially leading to dysregulation of genomic imprinting [34]. NAA15 mutations have also been described, with an overlapping phenotype to NAA10 mutations. Patients had several different indels or splice-site mutations, and presented with neurodevelopmental problems including intellectual disability, autism, motor function impairment and developmental delay [69]. Taken together, these findings suggest that NatA may have a critical role in nervous system development.

In this study, we present two male patients with a novel NAA10 variant, R83H. One patient presented with developmental delay, very limited language development at age 15, epilepsy, behavioural issues, but normal cardiac function. The other patient with a similar phenotype with developmental delay, very limited language development at age 12, ADHD like behaviour and hypertrophic cardiomyopathy. This is a similar clinical picture to what is known in some of the other described NAA10 patients with developmental delay and a cardiac phenotype.

Functional testing of in vitro catalytic activity was performed to determine whether the mutant enzyme was active. We found that NAA10-R83H had an unchanged substrate preference profile (Fig. 2a), but a greatly reduced catalytic activity. A time-course assay further bolstered this conclusion (Fig. 2c). The recurring R83C variant of NAA10 likewise had a greatly reduced catalytic activity [49], supporting the importance of R83 for NAA10 catalytic function.

We performed in silico mutagenesis to model the impact of this mutation on the surface charge of NAA10 (Fig. 3). While an arginine in this position contributes to a positively charged pocket, which can interact favorably with the phosphate groups on Ac-CoA, mutation to histidine decreases the positive charge density in this region. Several patients with a R83C variant at the same site have been reported. This variant likewise leads to a sharp decrease in catalytic activity [49]. The previously described R83C mutation and the loss of activity in R83H described here point to R83 being important for NAA10 enzymatic activity. The altered charge density in our in silico model suggests that perturbed interaction between NAA10-R83H and Ac-CoA may be the reason for the impaired catalytic activity. This is consistent with the hypothesis that the R83H variant leads to decreased NAA10 catalytic activity and that this is causing the symptoms observed in these two cases. Whether this is due to a loss of NAT- or KAT-activity, and if so which subset of substrates are mechanistically responsible for the phenotypes, is not clear.

## Conclusions

We identify a novel missense variant of NAA10, and present evidence to support that this variant is causative of the symptoms seen in the two cases.

## Additional file

Additional file 1: **Video S1.** Transthoracic echocardiography showing the left ventricle from an apical 3-chamber view (identical to Fig. 1) during two heart cycles in color compared mode. Please note that although there is no obstruction to flow in the left ventricular outflow tract (LVOT), a slight turbulence does occur along where the hypertrophic septum protrudes into the LVOT. (MOV 793 kb)

## Abbreviations

Ac-CoA: Acetyl coenzyme A
DD: Development delay
DNMT1: DNA methyltransferase 1
ID: Intellectual disability
IP: Immunoprecipitation
NAA10: N-alpha acetyltransferase 10
NAA15: N-alpha acetyltransferase 15
NAT: N-terminal acetyltransferase
NatA: N-terminal acetyltransferase A
PIX: p21-activated kinase-interacting exchange factor
WES: Whole exome sequencing
